# The London Lock Hospital

**Published:** 1902-05-03

**Authors:** 


### THE LONDON LOCK HOSPITAL.

### ANNUAL MEETING.

The Lord Kinnaird presided over the very small meeting of governors of this hospital on Thursday, the 24th of April, at the Male Hospital and Out-patients' Department, 91 Dean Street, Soho. Six gentlemen were present. The report, which was familiar to them all as members of the managing board, was taken as read. Lord Kinnaird ran over the principal points, and it was adopted unanimously. Everyone who visited the hospital, said the chairman, must share the feeling expressed in the opening words of the report, viz., of gratitude to Almighty God for the public sympathy and support during 1901. The work of the missions in connection with the hospital had been of great help and comfort to the patients, and the results went to prove that the work done by the honorary medical staff and the matron and nursing staff bore good fruit. They had been able to help 689 women, *i.e.*, 62 more than in the previous year. He was glad to be able to say that of those in the hospital 50 had passed into the Rescue Home, while 37 had been sent to other homes. The attendances of out-patients proved that the hospital was continuing to do the useful work it had done for many years past; the cause for its existence, he feared, was by no means diminishing. Thanks to the kindness of friends, the past year had been an exceptional one from a financial point of view, and over £2,000 was received in legacies. The first three months, however, of the present year showed that the need for support was as great as ever.

Mr. Parker Young seconded the motion. He said he thoroughly believed in the institution; it was doing a splendid work. He looked upon the Rescue Home as the best part of it; the women had been, in many cases, more sinned against than sinning. Ladies of his acquaintance held a very high opinion of the servants they obtained from the Home. A gentleman recently accused him of "pitching his money into a sink of iniquity;" that was the world's opinion.

At this point a governor remarked, "It's getting them out of the sink."

Thirty inmates, the speaker said, had been sent to service during the year. Any institution that dealt with sin and misery was unpopular, and this made it all the more necessary that money should be obtained from the various hospital funds. The Board most thoroughly appreciated the special donation of £750 from King Edward's Fund. What were they doing with the money? An immense amount. Anyone now visiting the wards would see that the patients had proper bedsteads, bedsteads that did not break the backs of the medical staff. A considerable amount more could be done if only they had more money. But when they finished up the year with a debt of nearly £900, these things were out of the question. He hoped they would be able to point out to the council of King Edward's Fund how matters stood. He frequently made surprise visits to the wards, and he always found the work satisfactory. What were the girls in the home taught? Sewing, washing, and cooking, and thus they turned them out efficient servants and respectable members of society. It was a God-like work, and commended itself to those who cared for rescue work. It could not be done without the hospital, where their bodies were first cured; it was in the home that they were led to higher things.

Mr. Tildesley enlarged on the value of the spiritual work done by the aid of the City Missionary and the lady who met the women out-patients weekly. The aim was to make those who had been a disgrace to society an ornament to it.

A vote of thanks to Lord Kinnaird, who represents the third generation of the family who have been closely connected with the hospital, brought the meeting to a close. Lord Kinnaird, in replying, said he hoped they did their medical work as well as any other hospital; they had also the important religious work carried on by the chaplain and the other workers alluded to.

